# Quadratus Lumborum Block Versus Transversus Abdominis Plane Block in Laparoscopic Colorectal Surgery: A Systematic Review and Meta-Analysis

**DOI:** 10.3390/medicina62010092

**Published:** 2026-01-01

**Authors:** Abdullah M. Alharran, Waleed Bader Alazemi, Saad A. Alajmi, Yousiff A. Bahman, Osamah Alhajri, Ali A. Alenezi, Jarrah J. Alenezi, Duaij Salman Saif

**Affiliations:** 1 Department of Surgery, College of Medicine and Medical Sciences, Arabian Gulf University, Manama 329, Bahrain; osamah_alhajri@outlook.com (O.A.); alialeneziemd@gmail.com (A.A.A.); jarrahalenezimd@gmail.com (J.J.A.); duaijsaif2323@gmail.com (D.S.S.); 2 Faculty of Medicine, University of Jordan, Amman 11942, Jordan; waleid990998@gmail.com; 3 Department of Surgery, Kuwait Institute for Medical Specializations, Kuwait City 12050, Kuwait; alajmi.s.a@gmail.com (S.A.A.); yousiffbahman@gmail.com (Y.A.B.)

**Keywords:** colectomy, rectal resection, analgesia, ERAS, anesthesia

## Abstract

*Background and Objectives*: Effective pain control after laparoscopic colorectal surgery is crucial for Enhanced Recovery After Surgery (ERAS) protocols. The transversus abdominis plane block (TAPB) provides somatic analgesia but lacks visceral coverage. The quadratus lumborum block (QLB) has emerged as an alternative, potentially offering both somatic and visceral blockade, but its superiority is debated. This systematic review and meta-analysis aimed to compare the analgesic efficacy of QLB versus TAPB in this setting. *Materials and Methods*: A comprehensive search of PubMed, Scopus, CENTRAL, and Web of Science was conducted for randomized controlled trials (RCTs) up to November 2025. Primary outcomes were 24 h postoperative and intraoperative opioid consumption. Secondary outcomes included pain scores, length of hospital stay (LoS), surgery duration, and adverse events. Standardized mean differences (SMD) and risk ratios (RR) were pooled. *Results*: Five RCTs involving 520 patients were included. No significant difference was found in 24 h postoperative opioid consumption (SMD: −1.62, 95% CI [−3.45, 0.20]; *p* = 0.08) or intraoperative opioid consumption (SMD: 0.38, 95% CI [−0.36, 1.12]; *p* = 0.31). QLB provided better, transient pain relief at rest at 12 h (SMD: −0.30, 95% CI [−0.52, −0.07]; *p* = 0.01) and during movement at 6 h (SMD: −0.20, 95% CI [−0.49, −0.09]; *p* = 0.01). No other time points for pain showed significant differences. QLB was associated with a shorter surgery duration (MD: −5.61 min, 95% CI [−10.38, −0.85]; *p* = 0.02), but not LoS (*p* = 0.53) or rates of PONV (*p* = 0.24) or dizziness (*p* = 0.32). *Conclusions*: With uncertain evidence, QLB and TAPB showed no significant difference in opioid consumption. QLB demonstrated a statistically significant but transient early analgesic advantage. This heterogeneity may be due to different QLB techniques, warranting further investigation.

## 1. Introduction

Laparoscopic colorectal surgery is a highly prevalent procedure [1]. Despite its minimally invasive nature, this surgery is associated with severe postoperative pain; hence, effective pain control during this procedure is a crucial component of Enhanced Recovery After Surgery (ERAS) protocols [2,3]. This pain is multifactorial, including a physical component from surgical cuts and port sites, in addition to a visceral component from peritoneal stretch and organ manipulation [4]. Poor pain management is a significant challenge to ERAS adherence, which is linked to increased opioid use [5], delayed return of normal bowel functions, delayed ambulation, and longer length of hospital stay (LoS) [6,7]. Therefore, effective, opioid-sparing analgesia is a critical component for optimizing patient recovery [8].

Multimodal analgesia (MMA) is the gold-standard approach to minimize opioid-related adverse events, with regional fascial plane blocks as the cornerstone of modern MMA [9,10,11]. The transversus abdominis plane block (TAPB) is a widely established and commonly used technique [12]. TAPB is recognized for its efficacy in providing somatic analgesia to the anterior abdominal wall, commonly affecting the T10-L1 dermatomes, depending on the approach used [13]. However, a significant limitation of the TAPB is its inability to offer consistent visceral analgesia [14], which is a key aspect of postoperative pain following colorectal surgery.

The quadratus lumborum block (QLB) has recently emerged as a promising alternative to the TAPB [15]. The QLB is suggested to provide a more comprehensive dermatomal block (T7-L1) and may offer visceral analgesia [16,17]. It is hypothesized that this visceral block is mediated by the diffusion of local anesthetic into the paravertebral space or via the thoracolumbar fascia [18]. Theoretically, the potential for a dual somatic-visceral block makes QLB a better option than TAPB for laparoscopic colorectal surgery.

Some randomized controlled trials (RCTs) have recently investigated QLB versus TAPB for laparoscopic colorectal surgery, but their findings are inconsistent [19,20,21,22,23]. Some studies [20,22] show a clear benefit for QLB, while others [21,23] show no significant difference in pain or opioid consumption. Therefore, the primary objective of this systematic review and meta-analysis is to compare the analgesic efficacy and safety of QLB versus TAPB in adult patients undergoing laparoscopic colorectal surgery.

## 2. Methodology

### 2.1. Protocol Registration

This systematic review was registered in PROSPERO [CRD420251233501]. The methods for this systematic review and meta-analysis complied with the Preferred Reporting Items for Systematic Reviews and Meta-Analyses (PRISMA) guidelines [24] and the Cochrane Handbook for Systematic Reviews of Interventions [25].

### 2.2. Data Sources and Search Strategy

A systematic literature search was conducted on 8 November 2025 by [A.M.A] across the following electronic databases: PubMed, Scopus, CENTRAL, and Web of Science. The search strategy utilized a combination of keywords and MeSH terms, including: (“quadratus lumborum block” OR QL block OR quadratus lumborum) AND (“transversus abdominis plane block” OR TAP block OR transversus abdominis) AND (colorectal OR rectal OR colectomy OR sigmoidectomy OR rectal resection) AND (laparoscop*). A complete overview of search terms and database results is presented in (Table S1). Additionally, we manually reviewed the reference sections of all eligible trials to guarantee comprehensive coverage and prevent the exclusion of any related studies.

### 2.3. Eligibility Criteria

RCTs were included if they followed the following Population, Intervention, Control, and Outcome (PICO) criteria:

- Population (P): adult patients undergoing elective laparoscopic colorectal surgery.
- Intervention (I): QLB, regardless of the approach, local anesthetic concentration, or volume.
- Control (C): TAPB, regardless of the approach, local anesthetic concentration, or volume.
- Outcomes (O): The primary outcomes were postoperative opioid consumption at 24 h and intraoperative opioid consumption. Secondary outcomes included pain scores at different postoperative time points at rest and during movement, length of hospital stay (LoS), surgery duration, and postoperative adverse events.

### 2.4. Study Selection

Two reviewers (W.B.A. and S.A.A.) independently assessed the eligibility of the retrieved records using Covidence. After the automated duplicate removal performed by Covidence, the remaining articles were screened across two phases. Initially, titles and abstracts were screened, and then the full texts of potentially eligible studies were assessed. Disagreement among reviewers was settled via discussion.

### 2.5. Data Extraction

Data extraction was independently performed by two reviewers (Y.A.B. and O.A). Any inconsistencies were resolved through discussion and consultation with the senior author. The data extraction process involved creating an Excel spreadsheet, which underwent pilot testing before formal extraction. The extraction form was organized into three main categories:

- Study characteristics: Study ID, country, study design, total number of patients, QLB details, TAPB group details, adjuvant analgesia, main inclusion criteria, pain assessment score, primary outcome, and follow-up duration.
- Participant baseline characteristics: number of participants in each group, age (years), gender (male/female), American Society of Anesthesiologists (ASA) classification, body mass index (BMI), and type of surgery.
- Outcome data: pain scores at all reported time points, total opioid/analgesic consumption, LoS, surgery duration, and postoperative adverse events.

Dichotomous data were extracted as the number of events and total participants, whereas continuous data were extracted as the mean and standard deviation. We utilized the formulas proposed by Wan et al. [26] to convert data reported as median and interquartile range into mean and standard deviation.

### 2.6. Risk of Bias and Certainty of Evidence

Methodological quality was evaluated for each RCT using the revised Cochrane Collaboration’s Risk of Bias tool (RoB 2) [27]. Two reviewers (A.A.A. and J.J.A.) independently assessed each study across the five domains (randomization process, deviations from intended interventions, missing outcome data, measurement of the outcome, and selection of the reported result). Disagreements were resolved by consensus. Additionally, the overall certainty of the evidence was assessed using the Grading of Recommendations Assessment, Development, and Evaluation (GRADE) approach [28,29], which considers risk of bias, inconsistency, indirectness, imprecision, and publication bias. Each factor was carefully considered, and the reasoning behind every decision was clearly explained, resolving differences through open discussion.

### 2.7. Statistical Analysis

The statistical analyses were performed using Stata/SE version 19.5 (StataCorp LLC, College Station, TX, USA). For continuous outcomes, Hedge’s g standardized mean difference (SMD) was calculated for pain and total opioid consumption, as studies used different assessment tools or units, respectively. Still, MD was used to pool surgery duration and LoS. The Risk Ratio (RR) was calculated for dichotomous outcomes. Heterogeneity was evaluated using the chi-squared test and the I^2^ statistic. A *p*-value less than 0.1 for the chi-squared test or an I^2^ value of 50% or higher indicated significant heterogeneity. Given the profound clinical heterogeneity arising from different QLB approaches and analgesic protocols, a random-effects model (REML) was prioritized for data synthesis to account for between-study variance. A fixed-effect model was utilized only for outcomes where statistical heterogeneity was negligible (I^2^ < 50%). In cases of significant heterogeneity, a leave-one-out sensitivity analysis was performed to investigate the stability of the results. Finally, an assessment of publication bias was not possible, as all analyzed outcomes included fewer than 10 RCTs [30].

## 3. Results

### 3.1. Search Results and Study Selection

**Figure 1:**
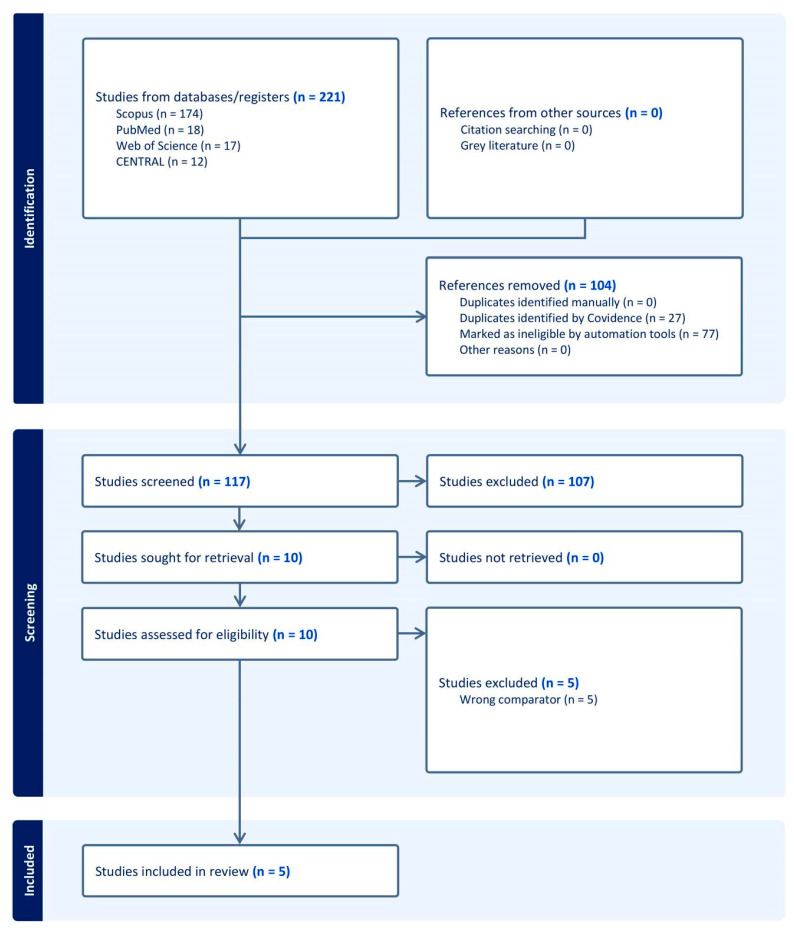
PRISMA flow chart of the screening process.

A total of 221 records were identified through database searching. After removing 104 irrelevant records, 117 records remained for screening. During the screening process, 107 records were excluded. Then, 10 full texts were assessed for eligibility. Of these, five studies were excluded for different reasons (Table S2). Finally, five trials [19,20,21,22,23] met the inclusion criteria and were included in the systematic review (Figure 1).

### 3.2. Characteristics of Included Studies

This review included five RCTs and 520 patients [19,20,21,22,23]. Four studies were conducted in China and one in the USA [21]. The QLB intervention consisted of a bilateral, single-shot, ultrasound-guided block; still, the specific technique differed between trials (either lateral, posterior, or posteromedial). Local anesthetic concentration ranged from 0.25% to 0.375% ropivacaine, with one study (George et al.) also including the adjuvant clonidine [21]. Further details on the study design of the included trials are outlined in (Table 1). Also, details on the included patients’ baseline data are outlined in (Table 2). Finally, complete details on the perioperative multimodal analgesia regimens and rescue protocols across the included studies are outlined in (Table S3).

### 3.3. Risk of Bias and Certainty of Evidence

**Figure 2:**
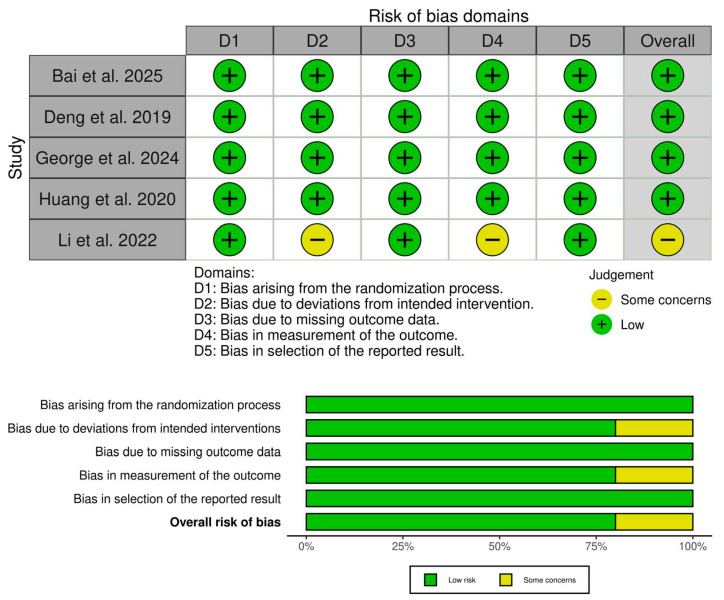
Quality assessment of risk of bias in the included trials [19,20,21,22,23]. The upper panel presents a schematic representation of risks (low = green, unclear = yellow, and high = red) for specific types of biases of the studies in the review. The lower panel presents risks (low = red, unclear = yellow, and high = red) for the subtypes of biases of the combination of studies included in this review.

Four RCTs showed an overall low risk of bias [19,20,21,22], and Li et al. showed some concerns [23] (Figure 2). The concerns mainly stemmed from the lack of information about blinding, leading to some concerns about performance and detection biases. Furthermore, our assessment of the certainty of evidence is highlighted in (Table 3).

### 3.4. Primary Outcome: Opioid Consumption

**Figure 3:**
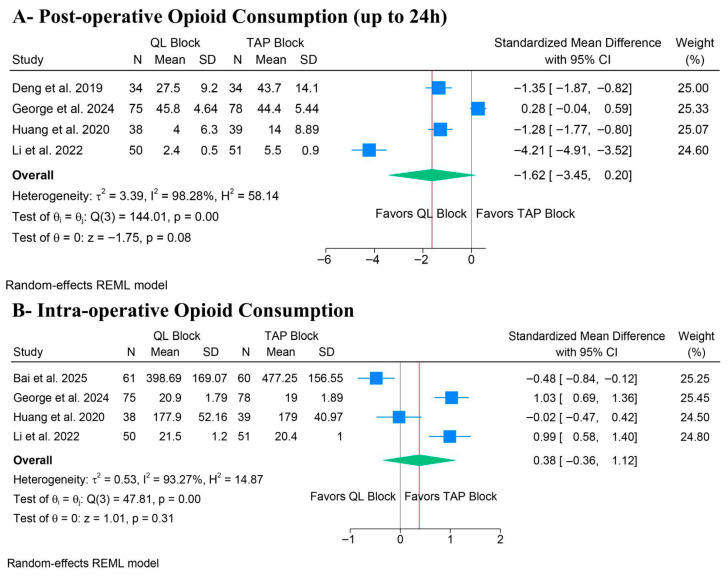
Forest plots of the primary outcomes (opioid consumption), CI: confidence interval [19,20,21,22,23].

There was no significant difference between QLB and TAPB groups regarding postoperative opioid consumption (SMD: −1.62, 95% CI [−3.45, 0.20], *p* = 0.08, I^2^ = 98.28%) (Figure 3A) and intra-operative opioid consumption (SMD: 0.38, 95% CI [−0.36, 1.12], *p* = 0.31, I^2^ = 93.27%) (Figure 3B). The stability of the primary outcomes was low. For post-operative opioid consumption, the leave-one-out sensitivity analysis revealed significant fragility in the pooled estimate. While the overall result was non-significant, the exclusion of a single study, George et al., shifted the result to statistical significance, favoring QLB (*p* = 0.018) (Figure S1). Additionally, the Galbraith plot revealed that George et al. and Li et al. are outliers and potentially contribute to the heterogeneity (Figure S2). Similarly, for intraoperative opioid consumption, the non-significant pooled estimate shifted to favor QLB after excluding Bai et al. (*p* = 0.047) (Figure S3). Additionally, the Galbraith plot revealed that George et al. and Bai et al. are outliers and potentially contribute to the heterogeneity (Figure S4). These sensitivity analyses indicate that the pooled results are heavily influenced by individual outliers utilizing the lateral QLB approach.

### 3.5. Secondary Outcomes

#### 3.5.1. Pain Score

##### Pain at Rest

**Figure 4:**
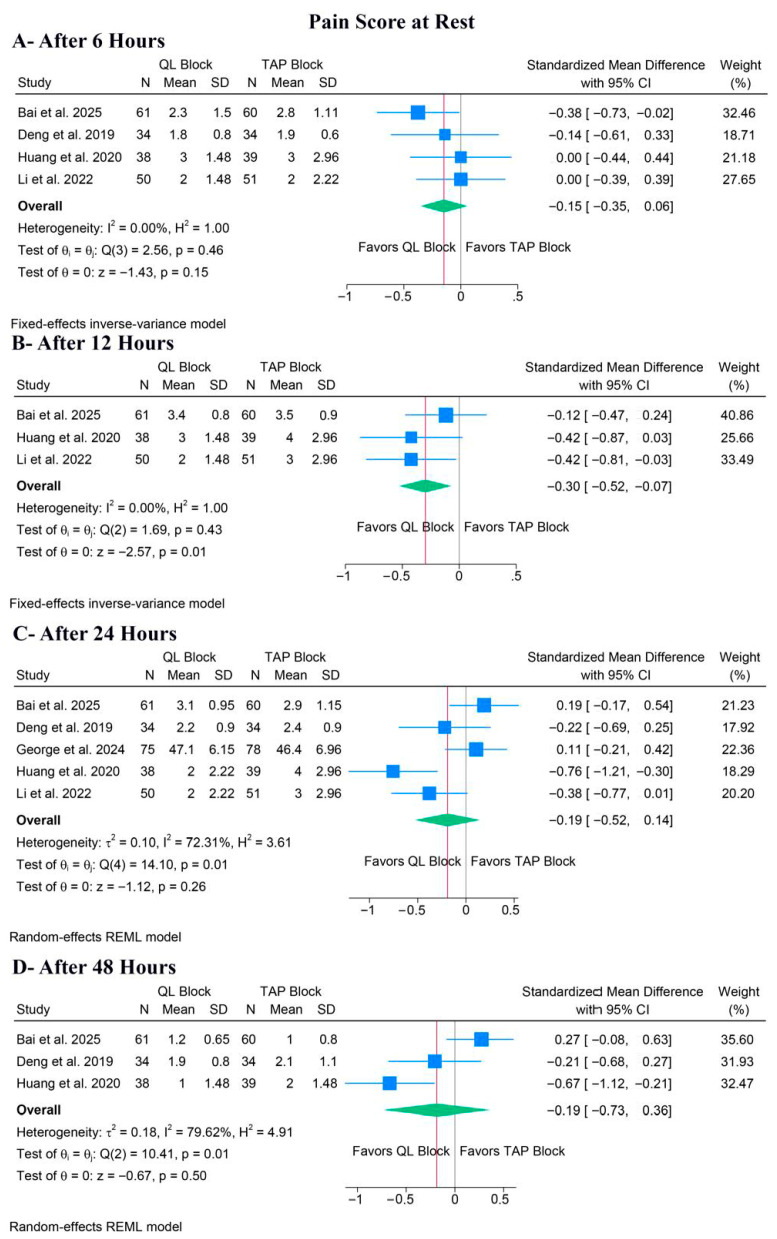
Forest plots of the secondary outcomes (pain score at rest), CI: confidence interval [19,20,21,22,23].

There was no significant difference between QLB and TAPB groups regarding pain at rest after 6 h (SMD: −0.15, 95% CI [−0.35, 0.06], *p* = 0.15, I^2^ = 0%) (Figure 4A), 24 h (SMD: −0.19, 95% CI [−0.52, 0.14], *p* = 0.26, I^2^ = 72.31%) (Figure 4C), or 48 h (SMD: −0.19, 95% CI [−0.73, 0.36], *p* = 0.50, I^2^ = 79.62%) (Figure 4D). Still, QLB significantly ameliorated pain at rest after 12 h (SMD: −0.30, 95% CI [−0.52, −0.07], *p* = 0.01, I^2^ = 0%) (Figure 4B).

For pain at rest after 24 h, a leave-one-out sensitivity analysis showed that the pooled estimate remained non-significant regardless of which study was omitted (Figure S5). Also, the Galbraith plot suggested that Huang et al. was a potential source of the observed heterogeneity (Figure S6). For pain at rest after 48 h, a leave-one-out sensitivity analysis showed that the pooled estimate remained non-significant regardless of which study was omitted (Figure S7). Also, the Galbraith plot suggested that Bai et al. was a potential source of the observed heterogeneity (Figure S8).

##### Pain During Movement

**Figure 5:**
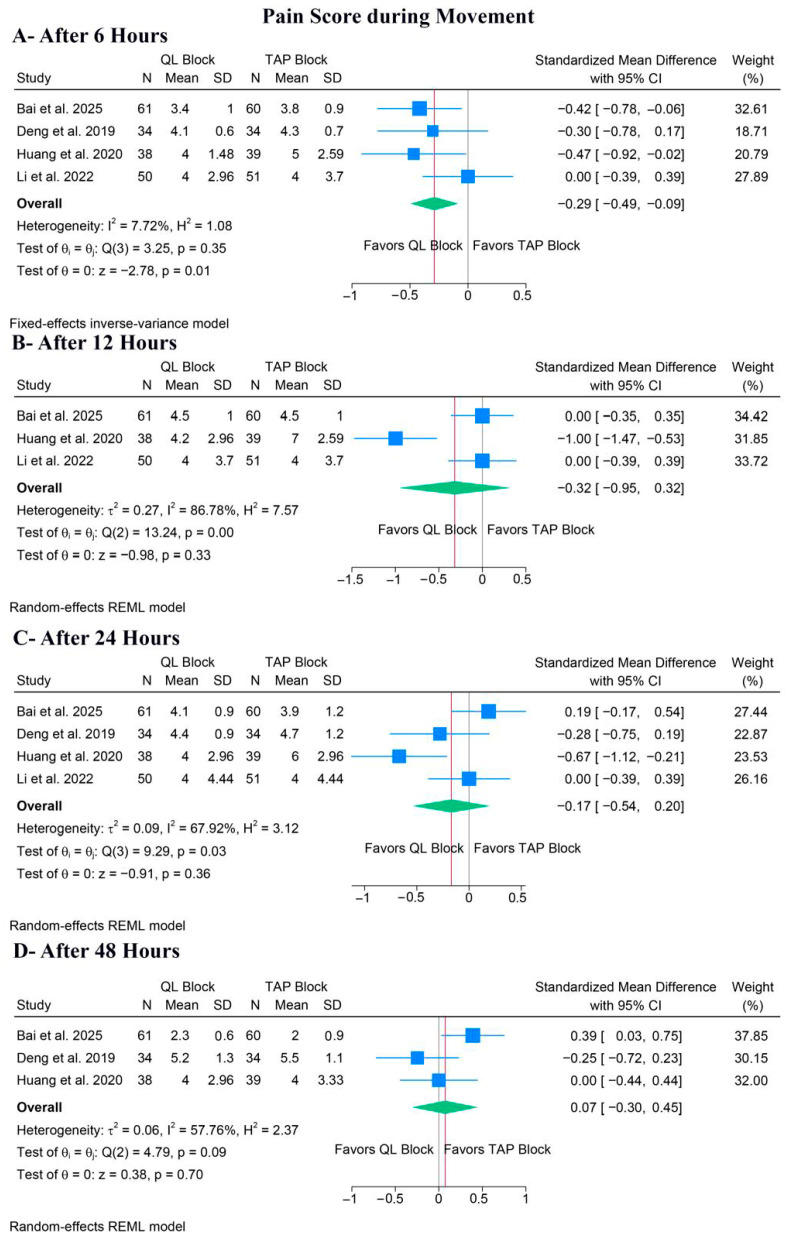
Forest plots of the secondary outcomes (pain score during movement), CI: confidence interval [19,20,22,23].

QLB significantly ameliorated pain during movement after 6 h (SMD: −0.20, 95% CI [−0.49, −0.09], *p* = 0.01, I^2^ = 7.72%) (Figure 5A); however, there was no difference between both groups after 12 h (SMD: −0.32, 95% CI [−0.95, 0.32], *p* = 0.33, I^2^ = 86.78%) (Figure 5B), 24 h (SMD: −0.17, 95% CI [−0.54, 0.20], *p* = 0.36, I^2^ = 67.92%) (Figure 5C), or 48 h (SMD: 0.07, 95% CI [−0.30, 0.45], *p* = 0.70, I^2^ = 57.76%) (Figure 5D).

For pain during movement after 12 h, a leave-one-out sensitivity analysis showed that the pooled estimate became statistically non-significant when either Bai et al. or Li et al. 2022 [23] was omitted (Figure S9). The corresponding Galbraith plot suggested that Huang et al. is a potential outlier and may be responsible for the observed heterogeneity (Figure S10). For pain during movement after 24 h, the leave-one-out sensitivity analysis showed that the pooled estimate remained non-significant when any study was omitted (Figure S11). The corresponding Galbraith plot suggested that Huang et al. is a potential outlier and may be responsible for the observed heterogeneity (Figure S12). Similar results were noted after 48 h (Figure S13), but the Galbraith plot showed no clear outliers (Figure S14).

#### 3.5.2. Surgery Duration and Hospital Stay

**Figure 6:**
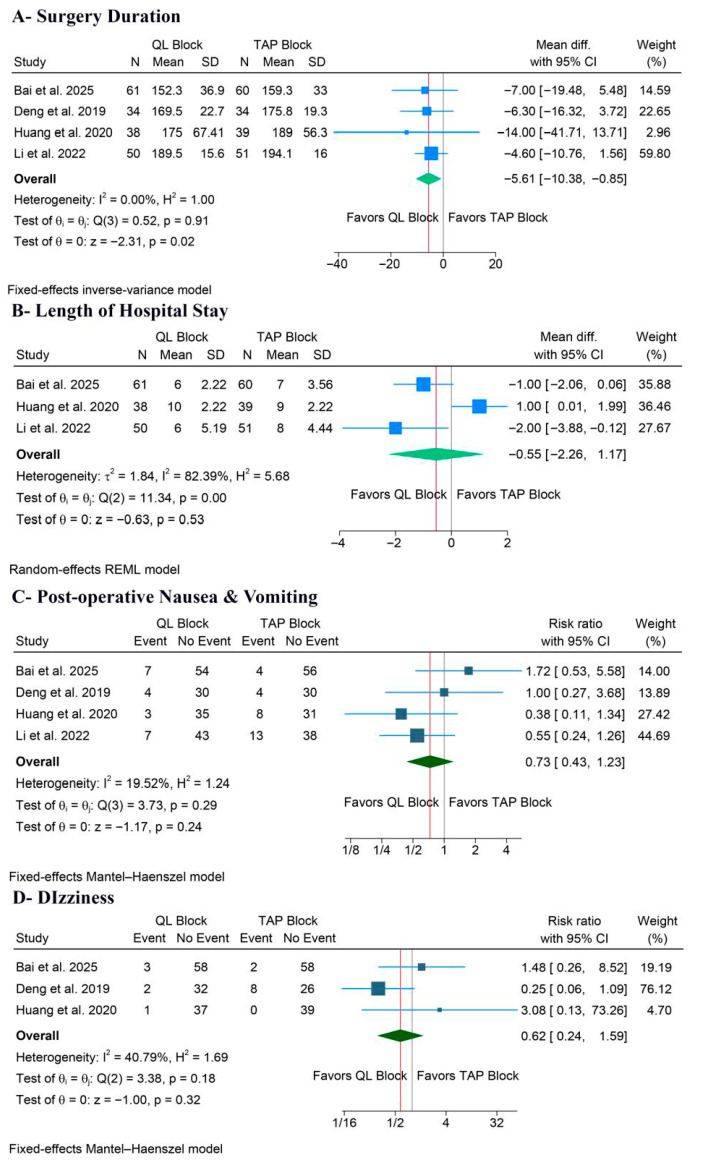
Forest plots of the other secondary outcomes, CI: confidence interval [19,20,22,23].

QLB significantly decreased surgery duration (MD: −5.61 min, 95% CI [−10.38, −0.85], *p* = 0.02, I^2^ = 0%) (Figure 6A). However, the LoS outcome showed no significant benefit for QLB compared to TAPB (MD: −0.55 days, 95% CI [−2.26, 1.17], *p* = 0.53, I^2^ = 82.39%) (Figure 6B). The leave-one-out sensitivity analysis showed that QLB was significantly associated with a shorter LoS after excluding the Huang et al. study (*p* = 0.008) (Figure S15). Also, the corresponding Galbraith plot suggested that Huang et al. is a potential outlier (Figure S16).

#### 3.5.3. Postoperative Adverse Events

There was no significant difference between the two block groups for either PONV (RR: 0.73, 95% CI [0.43, 1.23], *p* = 0.24, I^2^ = 19.52%) (Figure 6C) or dizziness (RR: 0.62, 95% CI [0.24, 1.59], *p* = 0.32, I^2^ = 40.79%) (Figure 6D).

## 4. Discussion

This systematic review and meta-analysis synthesized data from five RCTs involving 520 participants, yielding no statistically significant difference between QLB and TAPB for the primary outcomes of 24 h postoperative opioid consumption or intraoperative opioid consumption. For secondary outcomes, QLB was associated with a statistically significant, though transient, improvement in early pain scores, specifically pain during movement at 6 h and pain at rest at 12 h. However, the non-significant results for the primary outcomes were accompanied by high statistical heterogeneity. Also, QLB significantly decreased the surgery duration, with no difference in the LoS and adverse events, such as PONV and dizziness.

The QLB approach is potentially the main cause of the significant heterogeneity in most outcomes. This review pooled three anatomically and mechanistically distinct approaches: the lateral QLB (QLB type 1), used by Bai et al. and George et al.; the posterior QLB (QLB type 2), used by Deng et al. and Li et al.; and the posteromedial QLB (targeting the lumbar interfascial triangle), used by Huang et al. The lateral QLB is frequently regarded as a high somatic block, which is mechanistically comparable to a posterior TAPB [31]. Conversely, the posterior and posteromedial approaches involve injection posterior to the QL muscle, either into or close to the middle layer of the thoracolumbar fascia (TLF) [17]. This posterior positioning is hypothesized to facilitate the cephalad dissemination of local anesthetic to the thoracic paravertebral space and block sympathetic fibers within the TLF, potentially offering visceral analgesia [17].

While TAPB is well-established for managing somatic pain from the anterior abdominal wall [12,32], it inherently lacks the capacity to address the significant visceral pain component caused by peritoneal stretching and organ manipulation during colorectal surgery [4]. In contrast, posterior and posteromedial QLB approaches are hypothesized to facilitate the spread of local anesthetics into the paravertebral space or thoracolumbar fascia, thereby offering visceral blockade [33]. This mechanistic distinction is crucial for interpreting our results: the high heterogeneity likely stems from pooling studies that utilize the somatic-only lateral QLB (which functions similarly to TAPB) with those using posterior approaches that cover visceral pain [34]. Consequently, the distinct analgesic potential of posterior QLB is likely diluted when analyzed alongside the lateral approach.

Beyond the differences in block techniques, the heterogeneity in this meta-analysis may also be attributed to variations in the multimodal analgesia (MMA) regimens across the included trials. Comprehensive ERAS guidelines strongly recommend the scheduled use of paracetamol and NSAIDs to minimize opioid reliance [4,35]. In our review, only George et al. and Huang et al. utilized a robust MMA protocol that included scheduled paracetamol and NSAIDs [21,22]. In contrast, Bai et al., Deng et al., and Li et al. relied on opioid-based patient-controlled analgesia (PCA) without background paracetamol [19,20,23]. This difference in baseline non-opioid analgesia could alter the effect of the nerve block, as studies with less robust background analgesia might show a more pronounced benefit from the block. In contrast, rigorous MMA might mask the block’s benefit by lowering overall pain scores across both groups.

This hypothesis is strongly supported by a more in-depth analysis of the primary outcomes through sensitivity and outlier analyses. The overall pooled outcome for 24 h postoperative opioid consumption was not significant. However, it was considerably impacted by George et al., which favored TAPB and was identified as a significant outlier. Also, the leave-one-out sensitivity analysis confirmed that removing George et al. makes the pooled result statistically significant, favoring QLB. This finding can be explained by the following: first, it employed a lateral QLB, which, as argued, may be mechanistically inferior for this surgery compared to the posterior/posteromedial blocks used by other trials. Second, it used a different drug regimen (lower concentration 0.25% ropivacaine) with the adjuvant clonidine [32]. A similar pattern emerged for intraoperative opioid consumption. The non-significant result was dominated by the Bai et al. study, identified as an outlier; when removed, the findings were reversed, favoring QLB. Notably, Bai et al. also used the lateral QLB approach [19]. The high heterogeneity and sensitivity analyses provide compelling evidence that posterior QLB techniques are superior to TAPB. However, this advantage is statistically diminished when combined with the less effective lateral QLB studies.

In contrast to the primary outcomes, the analysis of early pain scores showed promising results favoring QLB, as QLB demonstrated a statistically significant benefit for pain during movement at 6 h and pain at rest at 12 h. Also, these findings were associated with low to no statistical heterogeneity, implying that all QLB approaches were superior to TAPB in this early phase. Despite this being an early and temporary advantage, it holds considerable significance in the context of ERAS protocols [8]. Current guidelines for modern colorectal ERAS protocols recommend early ambulation, frequently on the first postoperative day [35]. Optimizing pain relief within the initial 6–12 h, aligning with the maximal impact of a single-injection block, is crucial for promoting patient mobility, minimizing muscle guarding, and enhancing overall comfort [36]. The absence of a statistically significant difference at both 24 and 48 h is expected, as the resolution of the single-shot block effects occurs within 24 h, with pain management primarily controlled by the underlying multimodal analgesia protocol [16,32]. This result suggests that the overall 24 h opioid consumption measure may lack precision, as the considerable initial advantage is diluted by its inclusion of the 12–24 h period, during which the block’s effectiveness has diminished [37].

Moreover, QLB significantly decreased the surgery duration by a mean of 5.6 min, with no heterogeneity. However, this finding may not be clinically significant in long-lasting procedures, such as colorectal surgery, or may indicate a minor difference in block performance time [38]. Additionally, there was no difference for PONV. PONV and dizziness are primarily driven by opioid consumption. Given that the pooled 24 h opioid consumption was not significantly different, it is expected that these common opioid-related side effects would also be similar.

Additionally, although no events of local anesthetic systemic toxicity (LAST) were reported in the included trials, caution must be considered, and arrangements must be made to execute lipid resuscitation protocols if toxicity is suspected. To clarify, sufficient spread for fascial plane blocks, including QLB and TAPB, usually necessitates high volumes of local anesthetics, frequently leading to total doses approaching the maximum recommended levels [39]. Subsequently, these procedures carry a potential risk of local anesthetic systemic toxicity (LAST) due to systemic absorption [39,40]. Therefore, strict adherence to safety protocols is essential, and 20% lipid emulsion must be readily available whenever these blocks are performed, as it is the gold standard for treating LAST [41].

## 5. Implications for Clinical Practice

Based on the current evidence, the QLB, specifically the posterior approaches, appears to offer a clinical advantage over the TAPB for laparoscopic colorectal surgery due to its potential to provide visceral analgesia [17]. Posterior approaches can be considered as the preferred truncal block to support early recovery goals, such as early ambulation and reduced muscle guarding, particularly in the first 6–12 h post-surgery, where QLB showed superior pain relief. However, our results indicate that the analgesic benefit of the QLB is transient and does not significantly reduce cumulative opioid consumption at 24 h when pooled with lateral approach studies. Therefore, the QLB should not be the sole method for pain management in clinical practice. Instead, it must be integrated into a robust, guideline-compliant ERAS MMA that includes scheduled paracetamol and NSAIDs to bridge the analgesic gap once the block’s effect diminishes [35]. Future applications should also consider that the lateral QLB approach may perform similarly to a TAPB (somatic only) and might not justify the increased technical difficulty compared to TAPB if visceral coverage is not achieved [31].

## 6. Strengths and Limitations

Our review has several methodological strengths. It is, to the best of our knowledge, the first to synthesize RCTs comparing QLB versus TAPB in laparoscopic colorectal surgery. Additionally, we adhered strictly to the PRISMA guidelines, prospectively registered the protocol on PROSPERO, and included only high-quality RCTs, with four out of five judged to be at low risk of bias. Still, the main limitation of this review is the significant heterogeneity in data noted for most outcomes. The primary limitation of this review is the inability to perform a quantitative subgroup analysis stratified by the specific QLB technique (lateral, posterior, posteromedial). Although we identified the QLB approach as a significant source of clinical heterogeneity, the limited number of eligible RCTs resulted in subgroups containing only one or two studies for the primary outcomes. Such small sample sizes generate statistically unstable estimates and preclude a reliable head-to-head comparison of the different approaches. Consequently, as shown in the GRADE assessment, the certainty of evidence for all major outcomes was downgraded to low or very low.

## 7. Conclusions

Current evidence indicates no statistically significant difference between QLB and TAPB regarding opioid consumption and pain scores across most time points in patients undergoing laparoscopic colorectal surgery; however, these findings are limited by extreme statistical heterogeneity. This high variability is likely driven by the pooling of different QLB block techniques (lateral vs. posterior) and inconsistent MMA regimens across trials. Despite this statistical instability, QLB demonstrated a signal of efficacy for early pain relief (6–12 h) and reduced surgery duration compared to TAPB. Also, while posterior and posteromedial QLB approaches show promise for providing visceral analgesia, the high heterogeneity prevents a definitive conclusion regarding their overall superiority. Future research should prioritize head-to-head RCTs comparing specific QLB approaches (lateral vs. posterior) using standardized, guideline-compliant MMA protocols to resolve this clinical uncertainty.

## Figures and Tables

**Table 1:** Summary characteristics of the included RCTs.

Study ID	Study Design	Country	Total Participants	QLB Details	TAPB Details	Adjuvant Analgesia	Primary Outcome	Pain Assessment Score	Main Inclusion Criteria	Follow-Up Duration
Bai et al. 2025 [19]	RCT	China	121	Lateral QL block. 0.20 mL/side of 0.375% ropivacaine.	Bilateral TAP block. 0.20 mL/side of 0.375% ropivacaine.	Intraop: Remifentanil. Postop: IV sufentanil PCA, Flurbiprofen axetil (rescue).	Cumulative remifentanil administration during surgery.	VAS—0–10	18–80 years, ASA I-II, for elective laparoscopic radical resection (sigmoid or rectal).	48 h
Deng et al. 2019 [20]	RCT	China	68	Posterior QLB 0.20 mL/side of 0.375% ropivacaine.	Posterior TAPB 0.20 mL/side of 0.375% ropivacaine.	Intraop: Remifentanil, Sufentanil. Postop: IV parecoxib, Sufentanil PCIA (rescue).	Cumulative sufentanil consumption (at 6, 24, 48 h).	NRS—0–10	18–70 years, ASA I or II, for laparoscopic colorectal surgery.	48 h
George et al. 2024 [21]	RCT	USA	153	Lateral QL block 0.30 mL/side of 0.25% ropivacaine with 100 µg clonidine.	Bilateral TAP block 0.30 mL/side of 0.25% ropivacaine with 100 µg clonidine.	Postop: ERAS protocol (Acetaminophen, gabapentin, ketorolac). Rescue: Oral oxycodone, IV hydromorphone.	Dermatomal anesthetic spread.	VAS—0–100	≥18 years, for elective laparoscopic colorectal surgery	24 h
Huang et al. 2020 [22]	RCT	China	77	Posteromedial QL block (LIFT approach): 0.20 mL/side of 0.375% ropivacaine.	Lateral TAP block 0.20 mL/side of 0.375% ropivacaine.	Postop: Paracetamol (1 g q8h), Parecoxib (40 mg q12h). Rescue: Morphine PCA.	Cumulative morphine consumption 24 h postoperatively.	VAS—0–10	40–80 years, ASA I-II, for elective laparoscopic radical resection (colorectal cancer).	48 h (pain/opioids), 30 days (complications)
Li et al. 2022 [23]	RCT	China	101	QLB type 2 (Posterior approach). 0.20 mL/side of 0.375% ropivacaine.	Bilateral TAP block. 0.20 mL/side of 0.375% ropivacaine.	Intraop: Fentanyl, Remifentanil. Postop: Sufentanil PCA (with background infusion).	Quality of Recovery (QoR-15) scale at 24 h.	NRS—0–10	18–80 years, ASA I-III, for elective laparoscopic radical resection for rectal cancer.	24 h

QLB: Quadratus Lumborum Block; TAPB: Transversus Abdominis Plane Block; RCT: Randomized Controlled Trial; ASA: American Society of Anesthesiologists Physical Status Classification; VAS: Visual Analog Scale; NRS: Numeric Rating Scale; MME: Morphine Milligram Equivalents; PCA: Patient-Controlled Analgesia (used for intravenous administration); PCIA: Patient-Controlled Intravenous Analgesia (often synonymous with PCA); IV: Intravenous; LIFT: Lumbar Interfascial Triangle (specific injection site for posteromedial QLB); q8h: Every 8 h; q12h: Every 12 h; QoR-15: Quality of Recovery 15-item questionnaire; ERAS: Enhanced Recovery After Surgery (a multidisciplinary protocol).

**Table 2:** Baseline characteristics of the participants.

Study ID	Number of Participants in Each Group		Age (Years), Mean (SD)		Gender (Male/Female)		ASA I/II/III/IV		BMI, Mean (SD)		Type of Surgery	
	QLB	TAPB	QLB	TAPB	QLB	TAPB	QLB	TAPB	QLB	TAPB	QLB	TAPB
Bai et al. 2025 [19]	61	60	63 (53–67)	60 (51.3–67.8)	40 (65.6%)	39 (65.0%)	16/45/0	21/39/0	24.0 (3.1)	23.9 (3.0)	Rectal resection: 28; Sigmoid resection: 33	Rectal resection: 34; Sigmoid resection: 26
Deng et al. 2019 [20]	34	34	51.1 (13.8)	53.5 (10.6)	20 (58.8%)	22 (64.7%)	7/27/0	10/24/0	21.0 (6.4)	27.3 (7.3)	R. Hemicolectomy: 14; L. Hemicolectomy: 4; Ant. Resection: 10; Sigmoid colectomy: 4; Ileocolic resection: 2	R. Hemicolectomy: 16; L. Hemicolectomy: 5; Ant. Resection: 10; Sigmoid colectomy: 2; Ileocolic resection: 1
George et al. 2024 [21]	75	78	57.4 (15.2)	57.7 (16.7)	40 (53.3%)	38 (48.7%)	NR	NR	29.0 (6.3)	27.8 (6.0)	Colectomy w/ and w/o colostomy: 52; Enterostomy anastomosis/closure: 11; Bowel resection w/ ileostomy: 7; Other infraumbilical surgeries: 5	Colectomy w/ and w/o colostomy: 64; Enterostomy anastomosis/closure: 12; Bowel resection w/ ileostomy: 0; Other infraumbilical surgeries: 2
Huang et al. 2020 [22]	38	39	60.0 (10.8)	63.0 (7.5)	20 (52.6%)	22 (56.4%)	19/19/0	16/23/0	23.0 (3.1)	23.1 (3.4)	Low anterior resection: 24; Abd. perineal resection: 3; Right-side colonic resection: 9; Left-side colonic resection: 1; Subtotal colectomy: 1	Low anterior resection: 27; Abd. perineal resection: 2; Right-side colonic resection: 7; Left-side colonic resection: 3; Subtotal colectomy: 0
Li et al. 2022 [23]	50	51	67.9 (6.0)	68.0 (6.3)	23 (46.0%)	25 (49.0%)	3/30/17	4/32/15	23.1 (5.2)	23.5 (4.8)	Laparoscopic radical resection for rectal cancer	

QLB: Quadratus Lumborum Block; TAPB: Transversus Abdominis Plane Block; ASA: American Society of Anesthesiologists Physical Status Classification; BMI: Body Mass Index; SD: Standard Deviation; R. Hemicolectomy: Right Hemicolectomy; L. Hemicolectomy: Left Hemicolectomy; Ant. Resection: Anterior Resection; NR: Not Reported.

**Table 3:** GRADE evidence profile.

Certainty Assessment							Summary of Findings				
Participants (Studies) Follow-up	Risk of Bias	Inconsistency	Indirectness	Imprecision	Publication bias	Overall Certainty of Evidence	Study Event Rates (%)		Relative Effect (95% CI)	Anticipated Absolute Effects	
							With [TAPB]	With [QLB]		Risk with [TAPB]	Risk Difference with [QLB]
**Opioid Consumption (Postop up to 24 h)**											
397 (4 RCTs)	not serious	very serious ^a^	not serious	very serious ^b,c^	none	⨁◯◯◯ Very low ^a,b,c^	-	-	-	-	SMD **1.62 SD lower** (3.45 lower to 0.2 higher)
**Opioid Consumption (Intra-op)**											
452 (4 RCTs)	not serious	very serious ^a^	not serious	very serious ^b,c^	none	⨁◯◯◯ Very low ^a,b,c^	-	-	-	-	SMD **0.38 SD higher** (0.36 lower to 1.12 higher)
**Pain at Rest (6 h)**											
366 (4 RCTs)	not serious	not serious	not serious	Serious ^c^	none	⨁⨁⨁◯ Moderate ^c^	-	-	-	-	SMD **0.15 SD lower** (0.35 lower to 0.06 higher)
**Pain at Rest (12 h)**											
299 (3 RCTs)	not serious	not serious	not serious	Serious ^c^	none	⨁⨁⨁◯ Moderate ^c^	-	-	-	-	SMD **0.3 SD lower** (0.52 lower to 0.07 lower)
**Pain at Rest (24 h)**											
458 (5 RCTs)	not serious	Serious ^d^	not serious	very serious ^b,c^	none	⨁◯◯◯ Very low ^b,c,d^	-	-	-	-	SMD **0.19 SD lower** (0.52 lower to 0.14 higher)
**Pain at Rest (48 h)**											
299 (3 RCTs)	not serious	very serious ^a^	not serious	very serious ^b,c^	none	⨁◯◯◯ Very low ^a,b,c^	-	-	-	-	SMD **0.19 SD lower** (0.73 lower to 0.36 higher)
**Pain during Movement (6 h)**											
366 (4 RCTs)	not serious	not serious	not serious	Serious ^c^	none	⨁⨁⨁◯ Moderate ^c^	-	-	-	-	SMD **0.2 SD lower** (0.49 lower to 0.09 higher)
**Pain during Movement (12 h)**											
299 (3 RCTs)	not serious	very serious ^a^	not serious	very serious ^b,c^	none	⨁◯◯◯ Very low ^a,b,c^	-	-	-	-	SMD **0.32 SD lower** (0.95 lower to 0.32 higher)
**Pain during Movement (24 h)**											
366 (4 RCTs)	not serious	Serious ^d^	not serious	very serious ^b,c^	none	⨁◯◯◯ Very low ^b,c,d^	-	-	-	-	SMD **0.17 SD lower** (0.54 lower to 0.2 higher)
**Pain during Movement (48 h)**											
299 (3 RCTs)	not serious	Serious ^d^	not serious	Serious ^c^	none	⨁⨁◯◯ Low ^c,d^	-	-	-	-	SMD **0.07 SD higher** (0.3 lower to 0.45 higher)
**Surgery Duration (min)**											
366 (4 RCTs)	not serious	not serious	not serious	very serious ^b,c^	none	⨁⨁◯◯ Low ^b,c^	-	-	-	-	MD **5.61 min lower** (10.38 lower to 0.85 lower)
**Length of Hospital Stay (LoS)**											
299 (3 RCTs)	not serious	very serious ^a^	not serious	very serious ^b,c^	none	⨁◯◯◯ Very low ^a,b,c^	-	-	-	-	MD **0.55 days lower** (2.26 lower to 1.17 higher)
**PONV**											
366 (4 RCTs)	not serious	not serious	not serious	very serious ^b,e^	none	⨁⨁◯◯ Low ^b,e^	23/183 (12.6%)	16/183 (8.7%)	**RR 0.73** (0.43 to 1.23)	23/183 (12.6%)	**34 fewer per 1000** (from 72 fewer to 29 more)
**Dizziness**											
299 (3 RCTs)	not serious	not serious	not serious	very serious ^b,e^	none	⨁⨁◯◯ Low ^b,e^	10/150 (6.7%)	6/149 (4.0%)	**RR 0.62** (0.24 to 1.59)	10/150 (6.7%)	**25 fewer per 1000** (from 51 fewer to 39 more)

CI: confidence interval; MD: mean difference; RR: risk ratio; SMD: standardised mean difference; Explanations: ^a^. I^2^ > 75%. ^b^. A wide confidence interval. ^c^. Low number of participants. ^d^. I^2^ > 50%. ^e^. Low number of events.

## Data Availability

No new data were created or analyzed in this study.

## Supplementary Materials

The following supporting information can be downloaded at https://www.mdpi.com/article/10.3390/medicina62010092/s1. Table S1: Search strategy. Table S2: Excluded records in full-text screening. Table S3. Perioperative multimodal analgesia regimens and rescue protocols across the included studies. Figure S1: Leave-one-out sensitivity analysis for postoperative opioid consumption. Figure S2: Galbraith plot for postoperative opioid consumption. Figure S3: Leave-one-out sensitivity analysis for intraoperative opioid consumption. Figure S4: Galbraith plot for intraoperative opioid consumption. Figure S5: Leave-one-out sensitivity analysis for pain at rest at 24 h. Figure S6: Galbraith plot for pain at rest at 24 h. Figure S7: Leave-one-out sensitivity analysis for pain at rest at 48 h. Figure S8: Galbraith plot for pain at rest at 48 h. Figure S9: Leave-one-out sensitivity analysis for pain during movement at 12 h. Figure S10: Galbraith plot for pain during movement at 12 h. Figure S11: Leave-one-out sensitivity analysis for pain during movement at 24 h. Figure S12: Galbraith plot for pain during movement at 24 h. Figure S13: Leave-one-out sensitivity analysis for pain during movement at 48 h. Figure S14: Galbraith plot for pain during movement at 48 h. Figure S15: Leave-one-out sensitivity analysis for length of hospital stay (LoS). Figure S16: Galbraith plot for length of hospital stay (LoS). Table S1: Detailed electronic search strategy for all databases. Table S2: List of excluded studies with reasons for exclusion. Table S3: Perioperative multimodal analgesia regimens and rescue analgesia protocols across included studies.
