# Supplementary material for: Quadratus Lumborum Block Versus Transversus Abdominis Plane Block in Laparoscopic Colorectal Surgery: A Systematic Review and Meta-Analysis
Source: Medicina (Kaunas). 2026 Jan 1;62(1):92. doi: 10.3390/medicina62010092 (PMC12843055; doi:10.3390/medicina62010092)
Supplement: Supplementary file 1 [file medicina-62-00092-s001.zip › medicina-4022132-supplementary.pdf]

Supplementary Materials:

Contents:

Tables.

Table S1: Detailed electronic search strategy for all databases.
Table S2: List of excluded studies with reasons for exclusion.
Table S3. Perioperative multimodal analgesia regimens and rescue analgesia protocols across included studies.

Figures.

Figure S1: Leave-one-out sensitivity analysis of post-operative opioid consumption.
Figure S2: Galbraith plot of post-operative opioid consumption.
Figure S3: Leave-one-out sensitivity analysis of intra-operative opioid consumption.
Figure S4: Galbraith plot of intra-operative opioid consumption.
Figure S5: Leave-one-out sensitivity analysis of pain at rest after 24 hours.
Figure S6: Galbraith plot of pain at rest after 24 hours.
Figure S7: Leave-one-out sensitivity analysis of pain at rest after 48 hours.
Figure S8: Galbraith plot of pain at rest after 48 hours.
Figure S9: Leave-one-out sensitivity analysis for pain during movement at 12 h.

Figure S10: Galbraith plot for pain during movement at 12 h.

Figure S11: Leave-one-out sensitivity analysis for pain during movement at 24 h.

Figure S12: Galbraith plot for pain during movement at 24 h.

Figure S13: Leave-one-out sensitivity analysis for pain during movement at 48 h.

Figure S14: Galbraith plot for pain during movement at 48 h.

Figure S15: Leave-one-out sensitivity analysis for length of hospital stay (LoS).

Figure S16: Galbraith plot for length of hospital stay (LoS).

Table S1: *Detailed electronic search strategy for all databases*.

**Database**	**Search Terms**	**Search Field**	**Search Results**
PubMed	("quadratus lumborum block" OR QL block OR quadratus lumborum) AND ("transversus abdominis plane block" OR TAP block OR transversus abdominis) AND (colorectal OR rectal OR colectomy OR sigmoidectomy OR rectal resection) AND (laparoscop*)	All Fields	18
Cochrane	("quadratus lumborum block" OR QL block OR quadratus lumborum) AND ("transversus abdominis plane block" OR TAP block OR transversus abdominis) AND (colorectal OR rectal OR colectomy OR sigmoidectomy OR rectal resection) AND (laparoscop*)	All Text	12
WOS	("quadratus lumborum block" OR QL block OR quadratus lumborum) AND ("transversus abdominis plane block" OR TAP block OR transversus abdominis) AND (colorectal OR rectal OR colectomy OR sigmoidectomy OR rectal resection) AND (laparoscop*)	All Fields	17
SCOPUS	ALL (("quadratus lumborum block" OR QL block OR quadratus lumborum) AND ("transversus abdominis plane block" OR TAP block OR transversus abdominis) AND (colorectal OR rectal OR colectomy OR sigmoidectomy OR rectal resection) AND (laparoscop*))	All Fields	174

Table S2: List of excluded studies with reasons for exclusion.

Title	Published Year	DOI	Study ID	Exclusion Reason
Anterior quadratus lumborum block does not reduce postoperative opioid consumption following laparoscopic hemicolectomy: a randomized, double-blind, controlled trial in an ERAS setting	2022	10.1136/rapm-2022-103895	Tanggaard 2022	Wrong comparator
Ultrasound guided lateral quadratus lumborum block enhanced recovery in patients undergoing laparoscopic colorectal surgery	2021	10.1016/j.advms.2020.12.002	Wang 2021	Wrong comparator
Continuous basal infusion versus programmed intermittent bolus for quadratus lumborum block after laparoscopic colorectal surgery: a randomized-controlled, double-blind study	2020	10.1007/s00540-020-02791-x	Aoyama 2020	Wrong comparator
Effects of quadratus lumborum block regional anesthesia on postoperative pain after colorectal resection: a randomized controlled trial	2020	10.1007/s00464-019-07184-0	Boulianne 2020	Wrong comparator
Quadratus lumborum block versus perioperative intravenous lidocaine for postoperative pain control in patients undergoing laparoscopic colorectal surgery: A Prospective, Randomized, Double-blind Controlled Clinical Trial	2018	10.1097/SLA.0000000000002888	Dewinter 2018	Wrong comparator

Table S3. Perioperative multimodal analgesia regimens and rescue analgesia protocols across included studies.

Study ID	Intra-operative Adjuvants	Post-operative Analgesia	Rescue Analgesia	Paracetamol Used?
Bai et al. 2025	Atropine (0.5 mg)	Sufentanil PCA (2μg/kg in 100mL)/ Flurbiprofen axetil (50 mg) for rescue.	Flurbiprofen axetil (50 mg) if VAS ≥ 4	No
Deng et al. 2019	NR	Parecoxib (40 mg IV q12h)/ Sufentanil PCIA (no background infusion).	PCIA bolus (Sufentanil)	No
George et al. 2024	Multimodal analgesic regimen (discretion of anesthesiologist)	Acetaminophen/ Gabapentin/ Ketorolac (if age <75 & no renal disease)	Oxycodone (5 mg PO q4h). Hydromorphone (0.2-0.4 mg IV q3h)	Yes
Huang et al. 2020	Tropisetron (5 mg)/ Parecoxib (40 mg)	Paracetamol (1 g q8h)/ Parecoxib (40 mg q12h)/ Morphine PCA	Morphine PCA bolus	Yes
Li et al. 2022	NR	Sufentanil PCA (infusion 20μg/h + bolus)	Sufentanil PCA bolus	No

IV: Intravenous; NR: Not Reported; PCA: Patient-Controlled Analgesia; PCIA: Patient-Controlled Intravenous Analgesia; PO: Per Os (orally); VAS: Visual Analog Scale

- **IV:** Intravenous
- **NR:** Not Reported
- **PCA:** Patient-Controlled Analgesia
- **PCIA:** Patient-Controlled Intravenous Analgesia
- **PO:** Per Os (orally)
- **qXh:** Every X hours (e.g., q8h = every 8 hours; q12h = every 12 hours)
- **VAS:** Visual Analog Scale

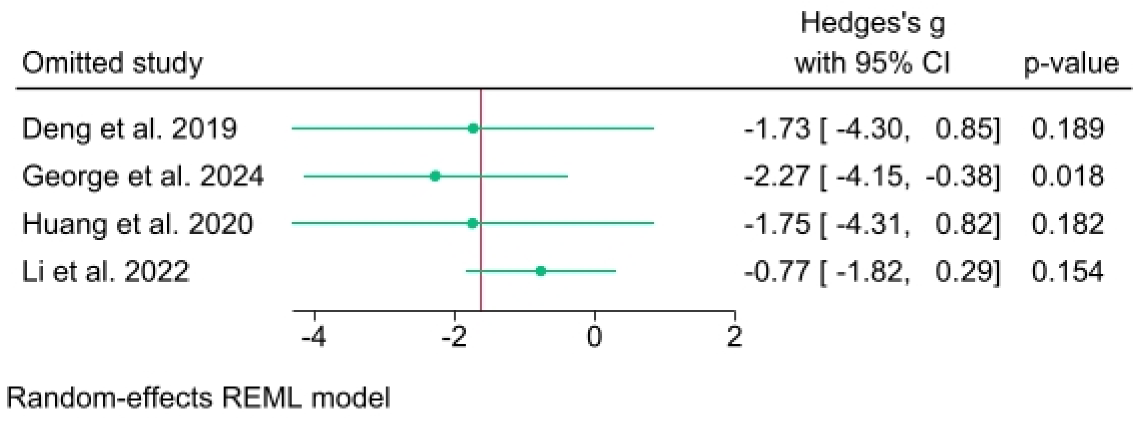

Figure S1: Leave-one-out sensitivity analysis of post-operative opioid consumption.

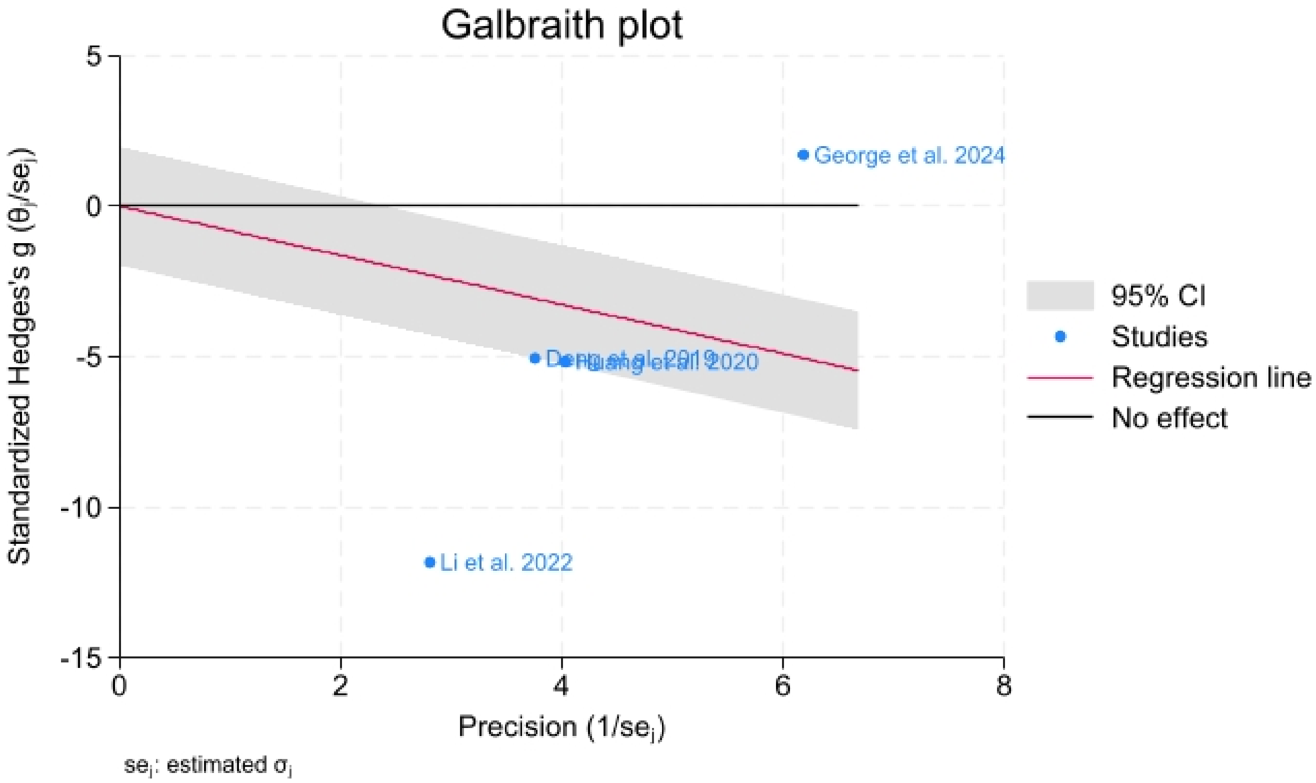

Figure S2: Galbraith plot of post-operative opioid consumption.

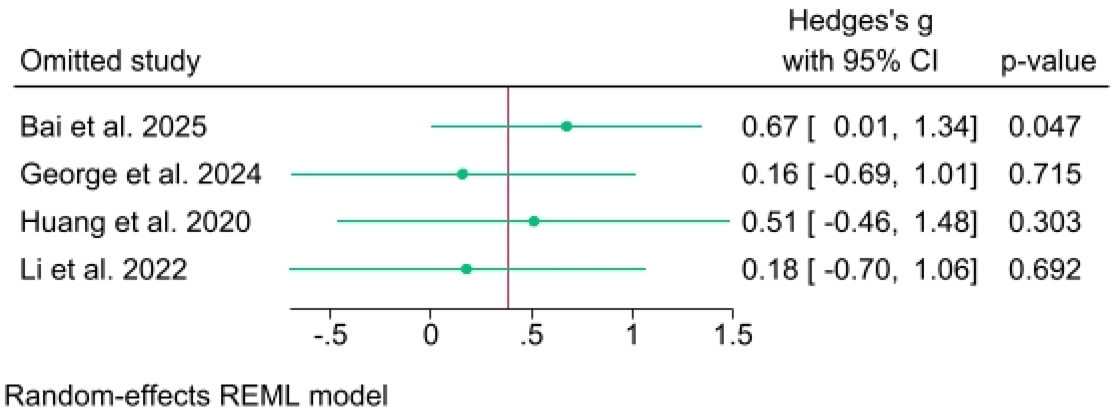

Figure S3: Leave-one-out sensitivity analysis of intra-operative opioid consumption.

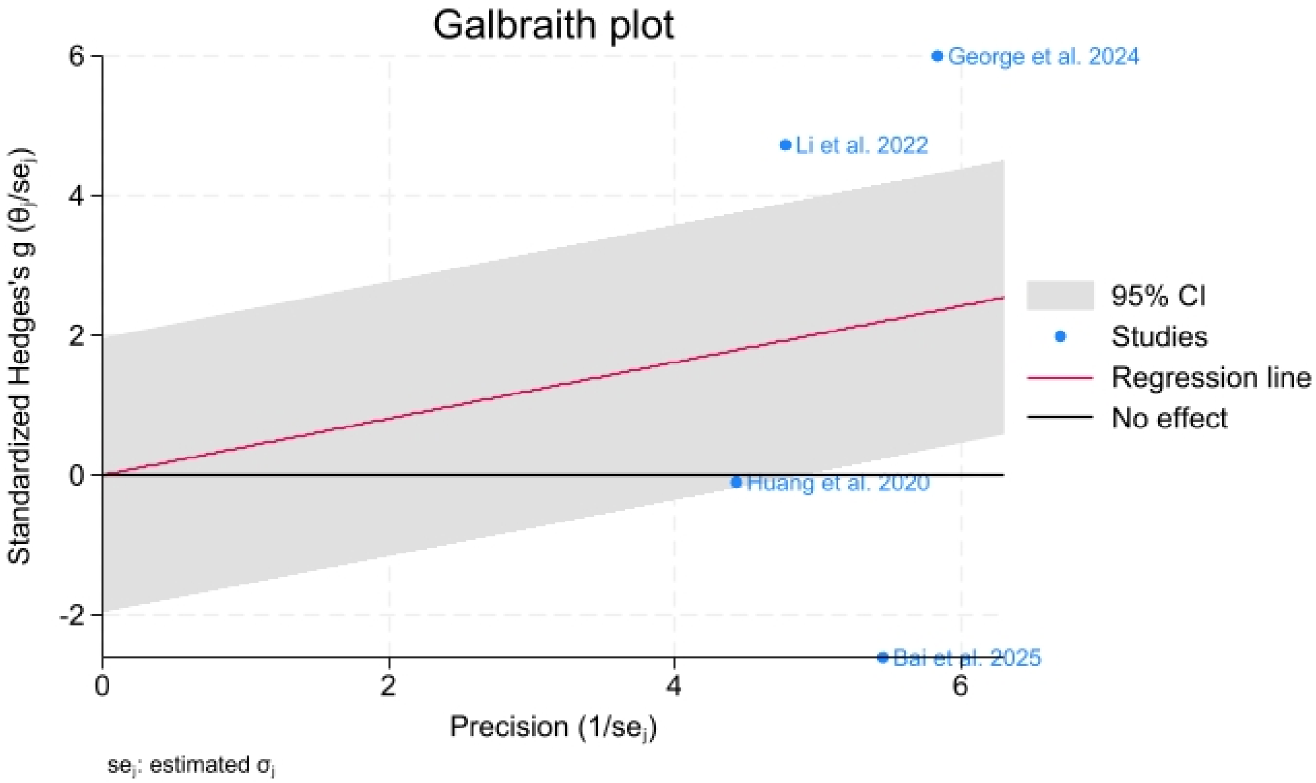

Figure S4: Galbraith plot of intra-operative opioid consumption.

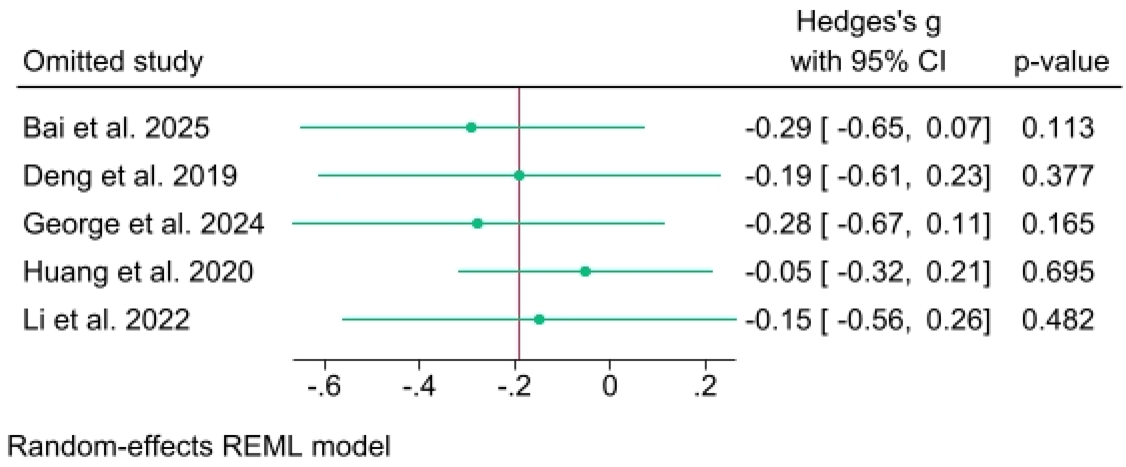

Figure: S5: Leave-one-out sensitivity analysis of pain at rest after 24 hours.

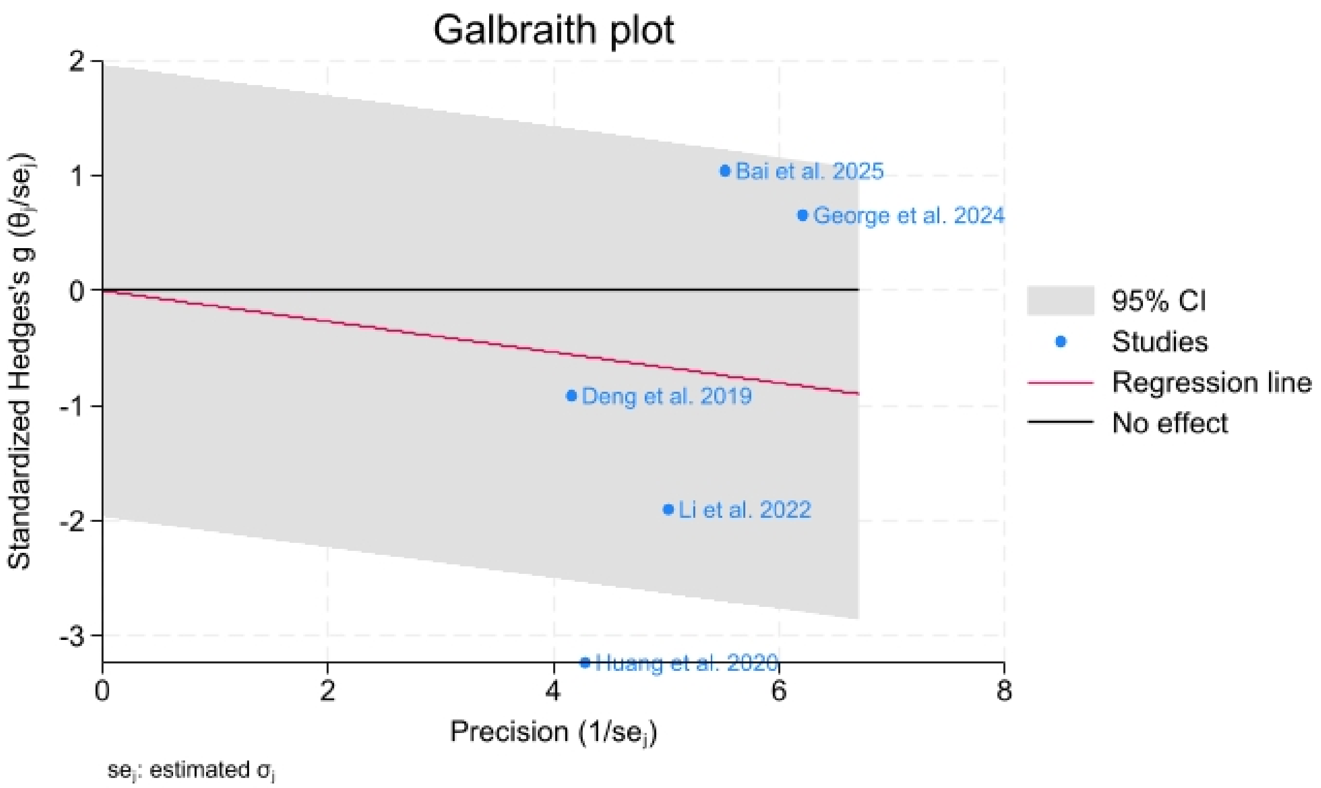

Figure S6: Galbraith plot of pain at rest after 24 hours.

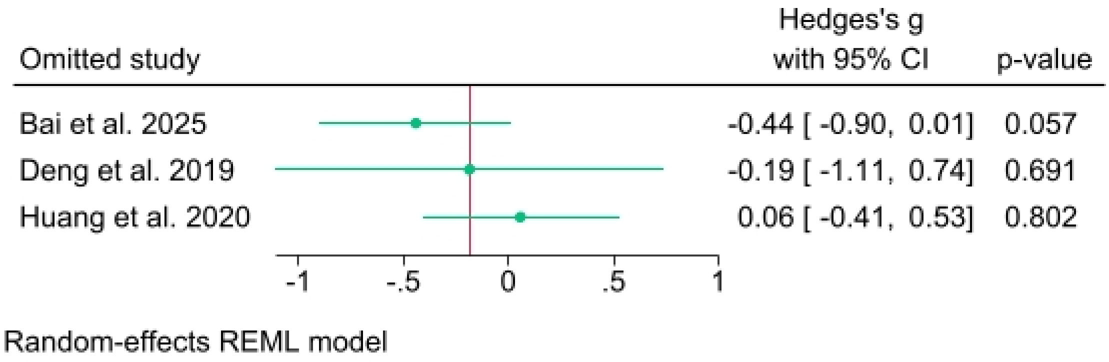

Figure S7: Leave-one-out sensitivity analysis of pain at rest after 48 hours.

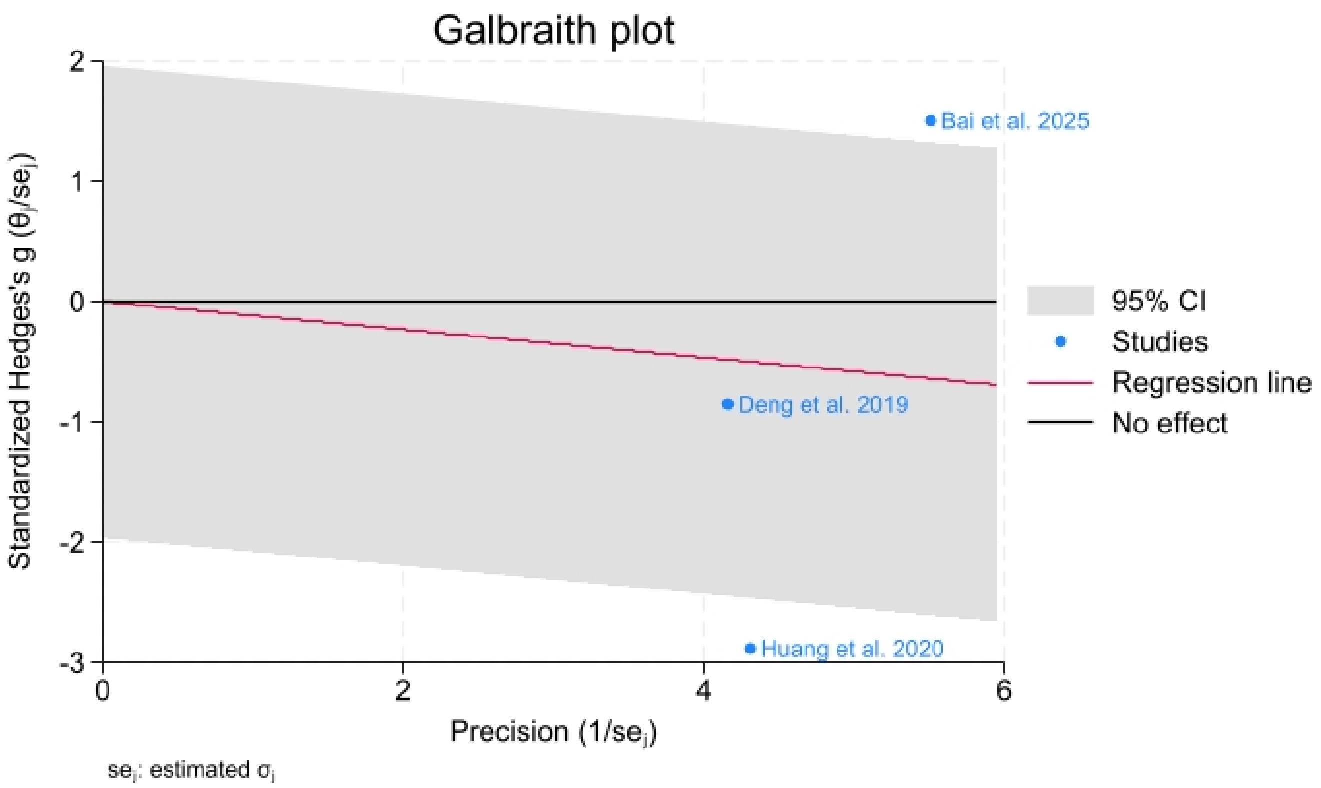

Figure S8: Galbraith plot of pain at rest after 48 hours.

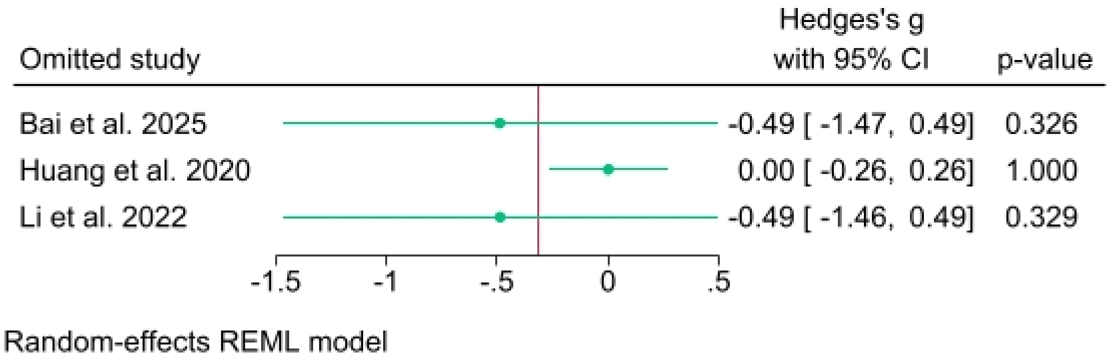

Figure S9: Leave-one-out sensitivity analysis for pain during movement at 12 h.

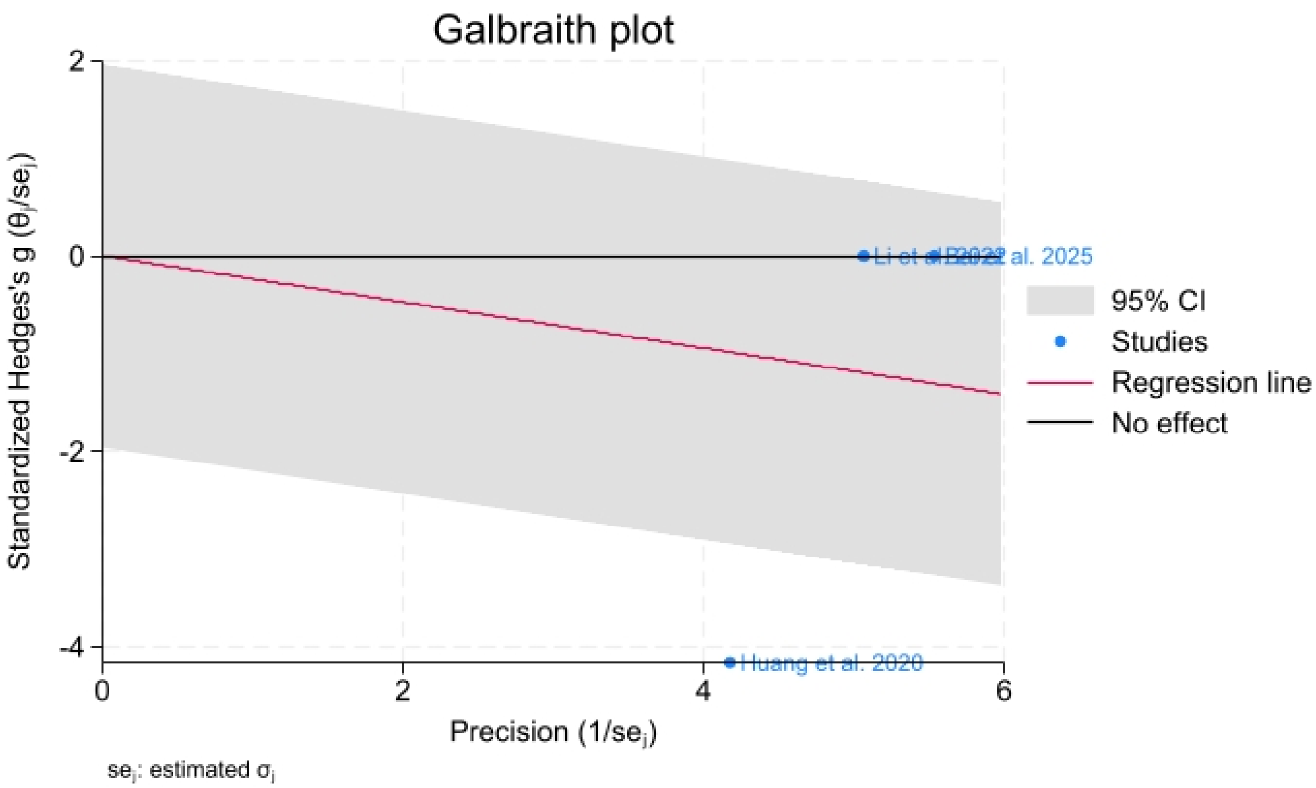

Figure S10: Galbraith plot for pain during movement at 12 h.

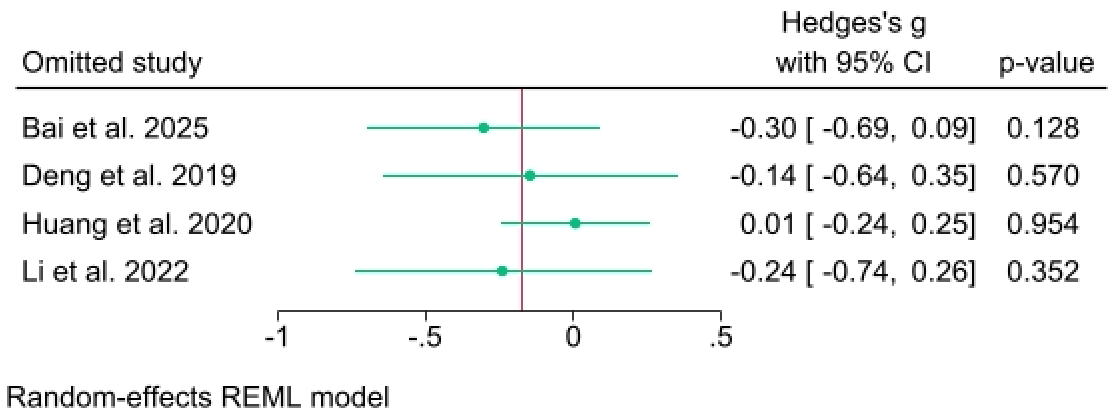

Figure S11: Leave-one-out sensitivity analysis for pain during movement at 24 h.

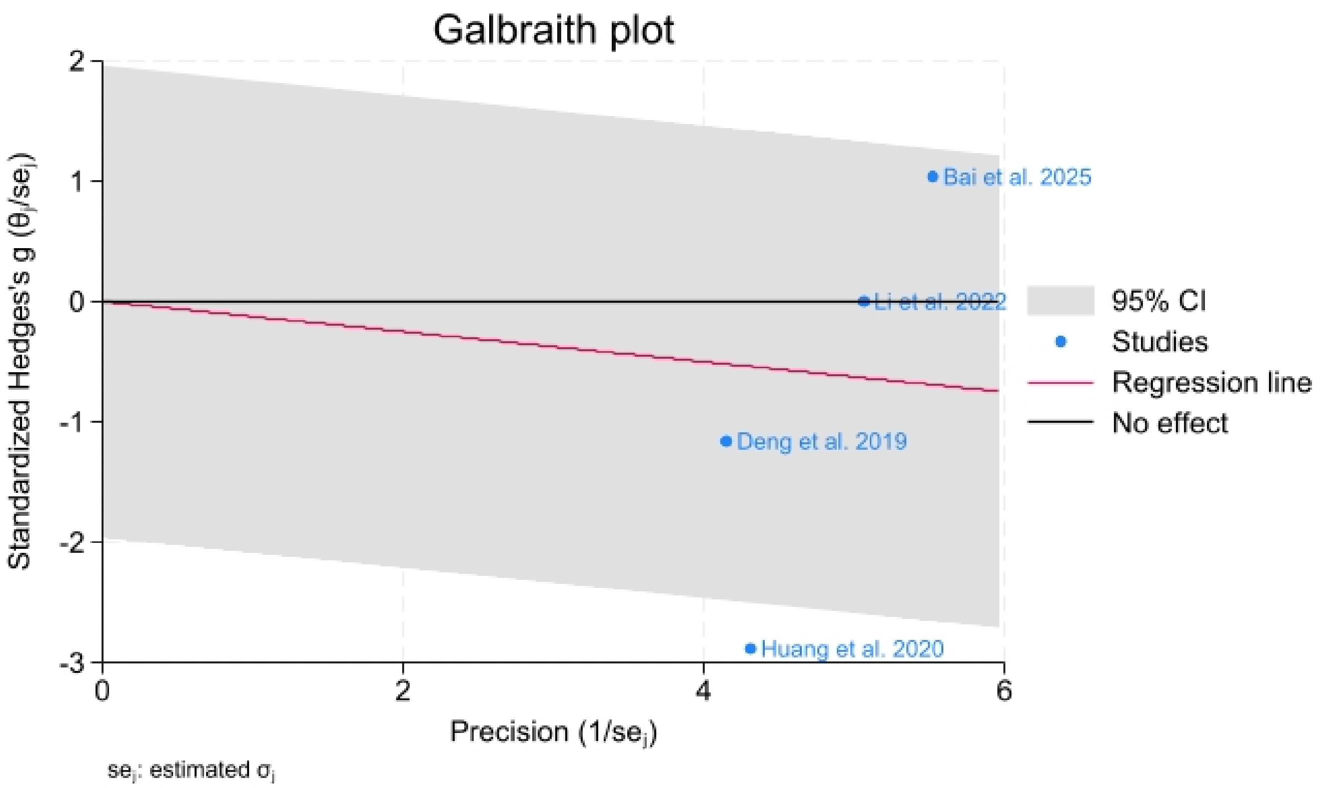

Figure S12: Galbraith plot for pain during movement at 24 h

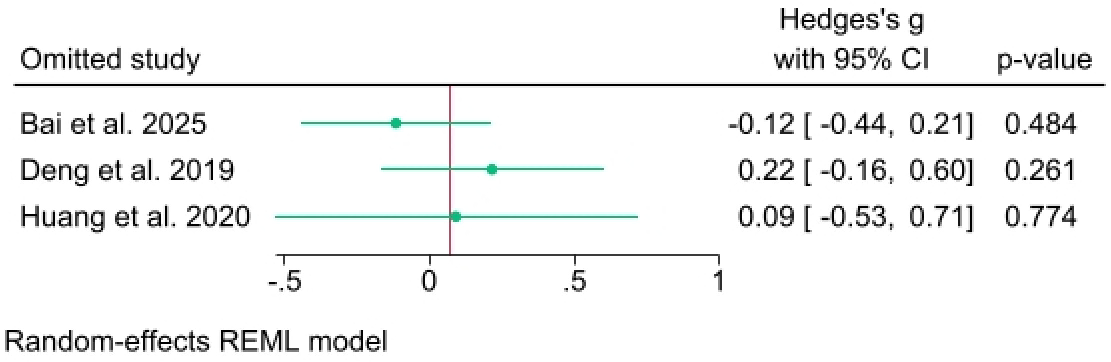

Figure S13: Leave-one-out sensitivity analysis for pain during movement at 48 h.

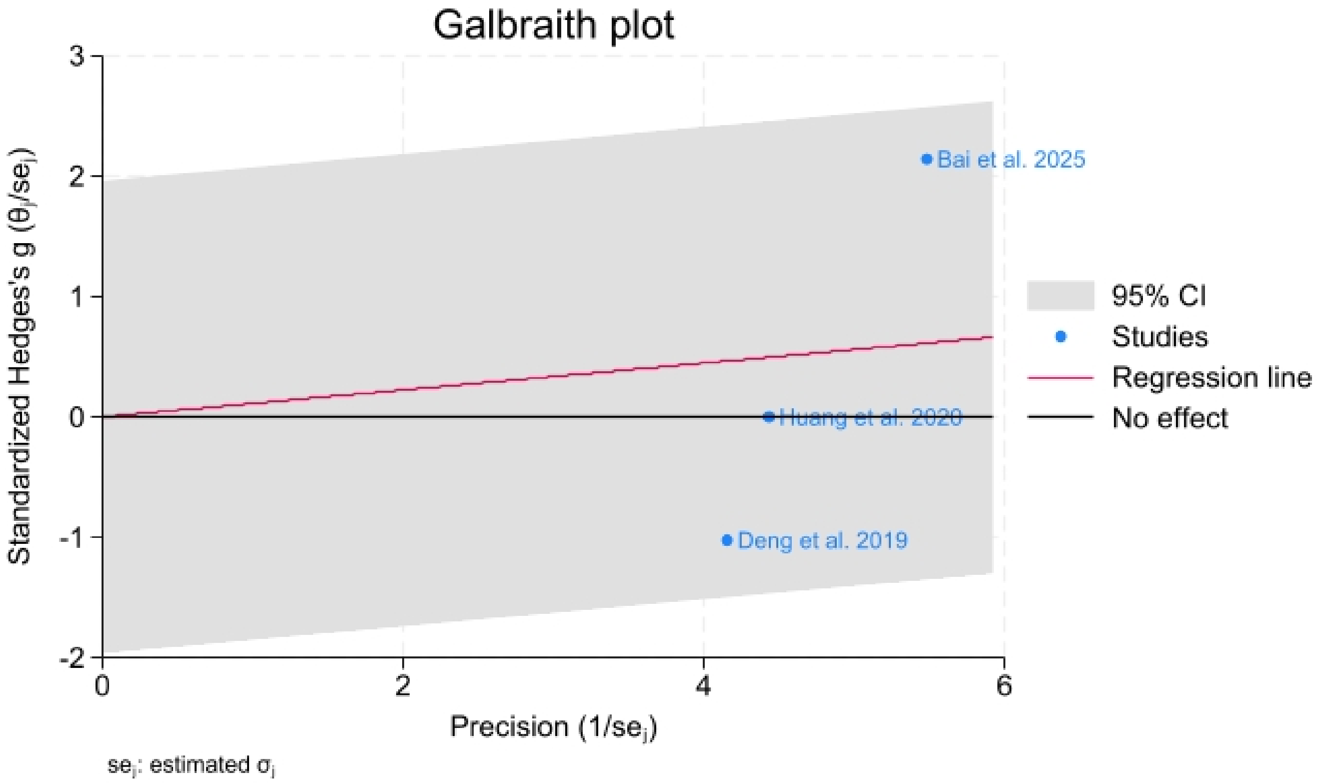

Figure S14: Galbraith plot for pain during movement at 48 h.

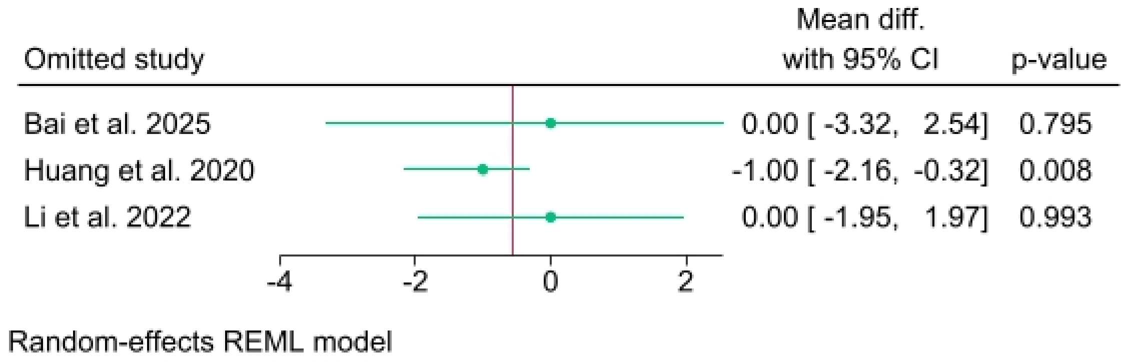

Figure S15: Leave-one-out sensitivity analysis for length of hospital stay (LoS).

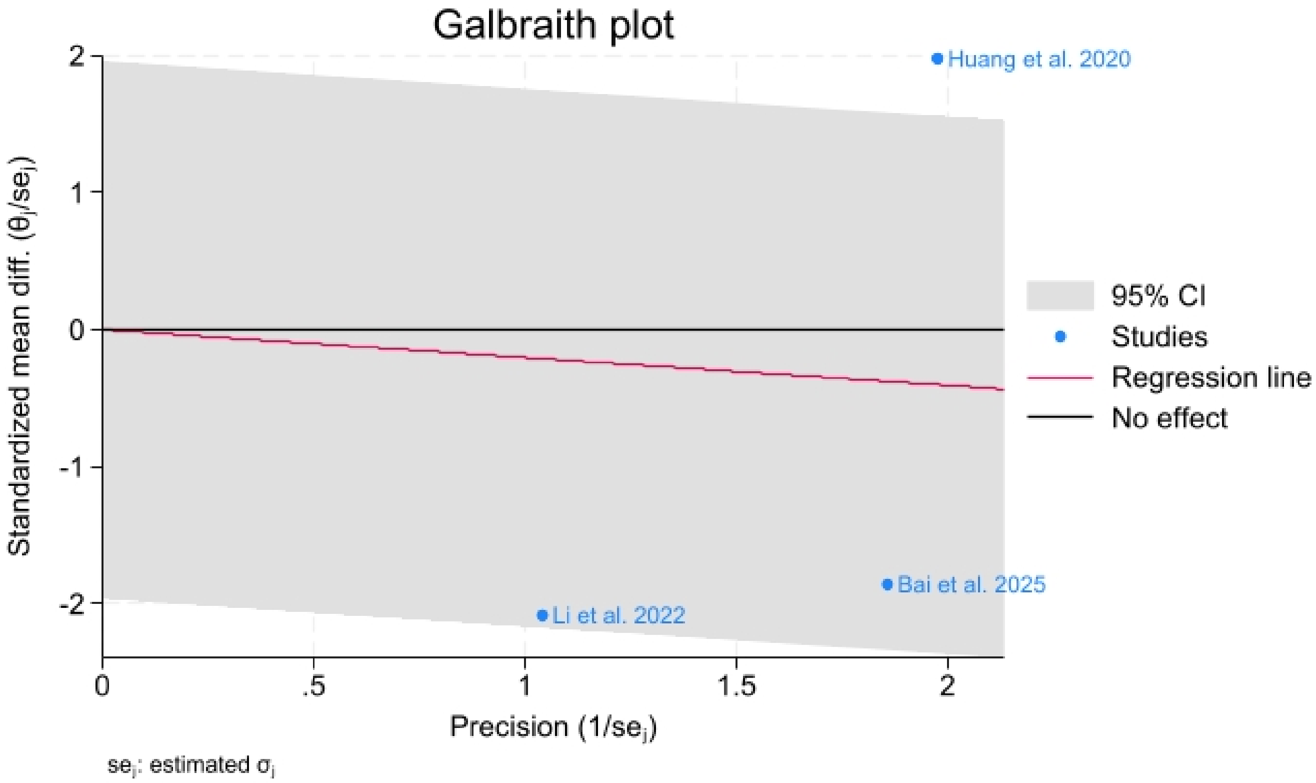

Figure S16: Galbraith plot for length of hospital stay (LoS).
